# Treatment of isolated distal deep vein thrombosis: an international survey of healthcare professionals

**DOI:** 10.1007/s11239-025-03091-5

**Published:** 2025-04-11

**Authors:** Ilia Makedonov, Lior Kapitanski, Joris Giai, Céline Vermorel, Elisabeth Chevrier, Grégoire Le Gal, Susan R. Kahn, Lina Khider, Marie-Antoinette Sevestre, Jameel Abdulrehman, Jean-Luc Bosson, Marc Righini, Jean-Philippe Galanaud

**Affiliations:** 1 Department of Medicine, Southlake Regional Health Centre, Newmarket and University of Toronto, Toronto, ON Canada; 2 Department of Medicine, Sunnybrook Health Sciences Centre, University of Toronto, Toronto, ON Canada; 3 Department of Public Health, University Grenoble-Alpes, CNRS, Grenoble-Alpes University Hospital, TIMC-IMAG, Grenoble, F38000 France; 4 Department of Vascular Medicine, St Philibert Hospital, Lomme, France; 5 Department of Medicine, The Ottawa Hospital and Hospital Research Institute, University of Ottawa, Ottawa, ON Canada; 6 Department of Medicine and Lady Davis Institute, Jewish General Hospital, Montreal, QC Canada; 7 Department of Vascular Medicine, European Hospital Georges Pompidou University Hospital, Paris, France; 8 Department of Vascular Medicine, Amiens University Hospital, Amiens, France; 9 Department of Medicine, University Health Network and University of Toronto, Toronto, ON Canada; 10 Department of Vascular Medicine, Geneva University Hospital, Geneva, Switzerland; 11 Division of Angiology and Hemostasis, Department of Medical Specialties, Faculty of Medicine, Geneva University Hospital, 4, rue Gabrielle-Perret-Gentil, Geneva 14, CH-1211 Switzerland

**Keywords:** Deep-vein thrombosis, Venous thrombosis, Ultrasound, Anticoagulation, Survey, Management

## Abstract

Isolated distal deep venous thrombosis (iDDVT) is an infra-popliteal DVT without pulmonary embolism (PE). It is a common condition, but its management remains debated. We conducted a survey distributed by email to the members of the French Society of Vascular Medicine, Thrombosis Canada, the Swiss Society of Angiology and INVENT-VTE research networks; Our objectives were to determine how healthcare professionals specialized in thrombosis manage iDDVT and to obtain experts input on the design of a future clinical trial. Participants were asked how they diagnosed and managed iDDVT and what should be the treatment arms of a future clinical trial on iDDVT management. 472 thrombosis specialists answered the questionnaire, mainly from France (*n* = 337), Canada (*n* = 61) and Switzerland (*n* = 28). Overall, 87.9% (*n* = 405) of respondents stated that their center always performed whole leg ultrasound in case of suspected DVT and that they managed patients with iDDVT usually at least once a week in 50.3% (*n* = 229) of cases. In 91.0% (*n* = 415) of cases respondents treated patients with iDDVT with anticoagulation more than 75% of time, with therapeutic doses and for a duration of 3 months in 90.2% (*n* = 406) and 74.7% (*n* = 334) of cases respectively. Most respondent managed muscular and deep-calf vein DVT similarly. More than 60% of respondents favored future trials comparing prophylactic versus therapeutic anticoagulation. Our real-world, international, practice survey of healthcare professionals shows that almost all respondents always conduct whole leg ultrasonography in case of suspected DVT and that they treat iDDVT with therapeutic anticoagulation usually for 3 months.

**Supplementary Information:**

The online version contains supplementary material available at 10.1007/s11239-025-03091-5.

## Key points

Management of isolated distal deep vein thrombosis (iDDVT) is controversial.

International survey assessing how healthcare professionals’ experts in thrombosis manage iDDVT.

When a DVT in the leg is suspected, most experts do whole leg ultrasound to screen deep veins of the calf.

Experts usually treat iDDVT with therapeutic anticoagulation for 3 months.

Future clinical trials on iDDVT should compare prophylactic vs. therapeutic anticoagulation.

**Supplementary Information:**

The online version contains supplementary material available at 10.1007/s11239-025-03091-5.

## Graphical abstract

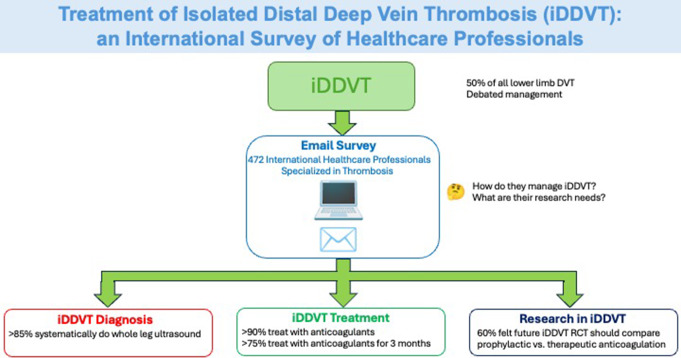

## Introduction

Isolated distal deep vein thromboses (iDDVT) are defined as infra-popliteal deep vein thromboses (DVT) without pulmonary embolism (PE). They represent more than half of all lower extremity DVT [1]. However, as compared to proximal DVT (DVT involving the popliteal or more proximal lower extremity deep veins) or PE, iDDVT have been poorly studied. Distal DVT has long been considered as a minor form of venous thromboembolism (VTE) that may not necessarily need to be either diagnosed or treated with anticoagulation [2]. Studies have confirmed that the risk of progression to fatal PE is exceptional with iDDVT [3]. However, the risks of DVT recurrence and post thrombotic syndrome are significant and could be reduced with the use of anticoagulants [4–6]. Nonetheless anticoagulant is not without harm and increases the risk of bleeding [7]. Several clinical trials on iDDVT have been conducted [3, 5]. These trials have compared varying intensities of anticoagulation (ranging from no anticoagulation/placebo ± compression stockings to therapeutic anticoagulation) and treatment durations (from 5 days to up to 6 months). Some studies have focused exclusively on muscular DVT; however, none have led to a consensus on the optimal approach. Thus, management of iDDVT is debated [7–10]. This uncertainty is reflected in current international guidelines. The American College of Chest Physicians suggests ultrasound surveillance and no treatment in cases of iDDVT without severe symptoms or risk factors for extension and to treat with anticoagulation otherwise [11]. The European Society of Cardiology makes a stronger statement in favor of anticoagulant treatment, stating that ‘patients with iDDVT at high-risk of recurrence should be anticoagulated, as for proximal DVT’, while for ‘those at low risk of recurrence shorter treatment, even at lower anticoagulant doses, or ultrasound surveillance may be considered’ [12]. The American Society of Hematology remains silent with the thorny problematics of iDDVT management [13].

The international RIETE registry showed that in routine clinical practice physicians generally treat iDDVT with anticoagulants, however controversy remains pertaining to the ideal dosing [14].

We conducted a survey among an international group of healthcare professionals with expertise in adult thrombosis medicine to assess how they usually manage iDDVT and to obtain their input regarding the design of a proposed clinical trial in iDDVT.

## Methods

An electronic survey was conducted among members of the French Society of Vascular medicine (SFMV), Thrombosis Canada, the Swizz Society of Angiology (SSA) and the International Network of VENous Thromboembolism (INVENT-VTE) research networks. The primary objective of this survey was to assess among healthcare professionals with expertise in Thrombosis how they usually managed iDDVT. The secondary objective was to obtain their input regarding the design of a proposed clinical trial in iDDVT. The study was approved by the local Institutional Review Board (IRB) at Sunnybrook Health Sciences Center (Ontario, Canada) (project identification number: 5675) on Feb 3, 2023.

### Study participants

Study participants were healthcare professionals with expertise in thrombosis (nurses, physicians and pharmacists) members of the INVENT-VTE network, Thrombosis Canada, SFMV and SSA societies. Administrative personnel in each organization were not eligible to participate. Healthcare professionals who were members of more than one participating organizations were asked to only complete one questionnaire.

### Electronic survey

An email inviting eligible health care professionals to participate was sent by the administrators of each organization to their members on February 6, 2023. The objective of the survey was explained and, for those who were willing to participate, there was a link to the online survey (done via LimeSurvey^®^). If the survey was not completed within the first 2 weeks of the invitation, a reminder email was sent. Data were collected anonymously. No information on sex, gender, ethnicity or age was collected.

### Study questionnaire

The questionnaire comprised of 8 questions on the management of iDDVT and 1 question on a proposed clinical trial on iDDVT (Additional Table 1).

**Table 1:** Diagnosis and management of isolated distal deep vein thrombosis (iDDVT) and preferred treatment arms if a future clinical trial on iDDVT management was to be conducted

	France *N* = 337	Canada *N* = 61	Switzerland *N* = 28	Other countries *N* = 46	Overall participants *N* = 472
**Use of whole leg ultrasound for suspected DVT**					
- Less than 50% of the time - Never - Sometimes but less than 50% of the time	0.9% (3) 0.3% (1) 0.6% (2)	31.6% (18) 10.5% (6) 21.1% (12)	0% (0) 0% (0) 0% (0)	6.8% (3) 4.5% (2) 2.3% (1)	5.2% (24) 2.0% (9) 3.3% (15)
- At least 50% of the time	1.5% (5)	26.3% (15)	17.9% (5)	15.9% (7)	6.9% (32)
- Always	97.6% (324)	42.1% (24)	82.1% (23)	77.3% (34)	87.9% (405)*
**Frequency of management of iDDVT** ^1^					
- Once every 3 months or less - Once every 3 months - Less than every 3 months	12.3% (40) 10.1% (33) 2.1% (7)	22.4% (13) 13.8% (8) 8.6% (5)	17.9% (5) 17.9% (5) 0% (0)	23.3% (10) 16.3% (7) 7.0% (3)	14.9% (68) 11.6% (53) 3.3% (15)
- At least once a month	30.7% (100)	50.0% (29)	46.4% (13)	37.2% (16)	34.7% (158)
- At least once a week	57.1% (186)	27.6% (16)	35.7% (10)	39.5% (17)	50.3% (229)*
**Treatment of iDDVT with anticoagulants** ^2^					
- Less than 50% of the time − 0–25% of cases − 26–50% of cases	1.8% (6) 0.9% (3) 0.9% (3)	15.5% (9) 8.6% (5) 6.9% (4)	0% (0) 0% (0) 0% (0)	4.7% (2) 2.3% (1) 2.3% (1)	3.7% (17) 2.0% (9) 1.8% (8)
− 51–75% of the time	1.8% (6)	15.5% (9)	14.3% (4)	11.6% (5)	5.3% (24)
- More than 75% of the time	96.3% (315)	69.0% (40)	85.7% (24)	83.7% (36)	91.0% (415)*
**Anticoagulant intensity** ^2^					
- Intermediate dose or less - Never treat with anticoagulants - Prophylactic doses of anticoagulants^3^ - Intermediate doses of anticoagulants^4^	1.6% (5) 0.3% (1) 0.9% (3) 0.3% (1)	8.6% (5) 3.4% (2) 3.4% (2) 1.7% (1)	0% (0) 0% (0) 0% (0) 0% (0)	7.1% (3) 0% (0) 2.4% (1) 4.8% (2)	2.9% (13) 0.7% (3) 1.3% (6) 0.9% (4)
- Therapeutic dose but without loading dose^5^	4.7% (15)	8.6% (5)	25.0% (7)	9.5% (4)	6.9% (31)
- Standard therapeutic dose^6^	93.8% (302)	82.8% (48)	75.0% (21)	83.3% (35)	90.2% (406)*
**Duration of anticoagulant treatment** ^2^					
- Less than 6 weeks	0.9% (3)	1.8% (1)	3.6% (1)	16.7% (7)	2.7% (12)
− 6 weeks	22.1% (71)	12.5% (7)	39.3% (11)	23.8% (10)	22.1% (99)
− 3 months or more − 3 months - more than 3 months	76.9% (247) 76.9% (247) 0% (0)	85.7% (48) 83.9% (47) 1.8% (1)	57.1% (16) 57.1% (16) 0% (0)	59.5% (25) 57.1% (24) 2.4% (1)	75.2% (336)* 74.7% (334) 0.4% (2)
**Treatment of Muscular vein thrombosis (comparatively to deep calf vein DVT)** ^2^					
- Same treatment	80.9% (258)	73.1% (38)	78.6% (22)	85.4% (35)	80.2% (353)
- Different treatment, consisting of:					
- No anticoagulation - Shorter duration of anticoagulation - Lower doses of anticoagulant - Shorter duration and lower doses of anticoagulants	0.6% (2) 16.0% (51) 0.3% (1) 2.2% (7)	11.5% (6) 3.8% (2) 7.7% (4) 3.8% (2)	0% (0) 3.6% (1) 7.1% (2) 10.7% (3)	4.9% (2) 2.4% (1) 2.4% (1) 4.9% (2)	2.3% (10) 12.5% (55) 1.8% (8) 3.2% (14)
**Preferred treatment arms if a future clinical trial on iDDVT management**					
- Placebo/no AC vs. Prophylactic AC	3.7% (12)	6.7% (4)	7.1% (2)	9.8% (4)	4.9% (22)
- Placebo/no AC vs. Therapeutic AC	27.4% (88)	21.7% (13)	10.7% (3)	24.4% (10)	25.3% (114)
- Prophylactic AC vs. Therapeutic AC	60.7% (195)	63.3% (38)	75.0% (21)	53.7% (22)	61.3% (276)
- Other	8.1% (26)	8.3% (5)	7.1% (2)	12.2% (5)	8.4% (38)

*p < 0.01

^1^ Among those who manage iDDVT; ^2^ Among those who treat iDDVT; ^3^ Prophylactic doses: apixaban 2.5 mg BID, rivaroxaban 10 mg daily, enoxaparin 40 mg sc daily …; ^4^ Intermediate doses: higher than prophylactic doses and lower than therapeutic doses, rivaroxaban 15 mg daily, enoxaparin 1 mg/Kg sc daily etc…; ^5^ Therapeutic doses of DOAC, but without initial higher dose apixaban 10 mg BID for first 7 days or rivaroxaban 15 mg BID for first 21 days if apixaban or rivaroxaban used, or without initial 5–7 days of parenteral anticoagulation if edoxaban or dabigatran used; ^6^ Standard therapeutic doses: same doses as those used for patients with proximal DVT and /or PE

˜

### Outcomes measures

The main outcome measure was the proportion of healthcare professionals who treated iDDVT with anticoagulants and at which dose (therapeutic, intermediate or prophylactic doses); The secondary outcome was the proposed management arms for future clinical trials.

### Statistical analysis

Data analysis consisted of standard descriptive analyses of qualitative data, with numbers and mean. For each survey question, Chi-squared (or Fisher exact) tests were used to determine whether the proportions of replies were different between countries (France, Canada, Switzerland and other countries). Two sided *p*-values of 0.05 or less were considered to be statistically significant. Data were analyzed using STATA, version 17.0.

## Results and discussion

A total of 472 healthcare professionals completed the survey. Most respondents (*n* = 426, 90.3%) were from French (*n* = 337), Canadian (*n* = 61) and Swiss (*n* = 28) organizations. Other respondents (*n* = 46, 9.7%) were mainly from Australia or New Zealand (*n* = 25) and the USA (*n* = 13).

The main results of the survey are presented in Table 1.

In case of suspected DVT, a whole leg US is always performed in 87.9% (*n* = 405) of participants institutions. This proportion is consistent among participants from the various countries with the exception of Canada where whole leg US is always performed in only 42.1% of institutions, *p* < 0.001.

As a consequence, Canadian healthcare professionals are likely less exposed to iDDVT. While half (50.3% *n* = 229) of survey respondents reported that they manage iDDVT at least once a week, only a quarter (27.6%, *n* = 16) of Canadian healthcare professionals manage iDDVT once a week (*p* < 0.001), and half of them manage iDDVT once a month.

In line with data from the RIETE registry, in the absence of significant bleeding risk, 91.0% (*n* = 415) of respondents stated that they always treat iDDVT with standard therapeutic doses of anticoagulants (90.2%, *n* = 406) for a 3 month period (74.7%, *n* = 334) [15]. This practice is supported by data from two recently published Italian RCTs, which showed that patients with iDDVT treated for 3 months with anticoagulation had lower risks of VTE recurrence than those treated for shorter durations [16, 17].

In line with the finding that whole leg US are conducted less often in Canada for suspected DVT, iDDVT are also less likely to be always treated with anticoagulants in Canada than in other countries (69.0% (*n* = 40) vs. 94.2% (*n* = 375), *p* < 0.001). However, when IDDVT are treated with anticoagulants this is usually with ‘standard’ full therapeutic doses. Interestingly, there is a trend among Swiss respondents, compared to other countries respondents, towards more frequently treating iDDVT with therapeutic anticoagulation without initial loading dose when apixaban or rivaroxaban are used or without initial 5–7 days of parenteral anticoagulation if edoxaban or dabigatran used (25.0% (*n* = 7) vs. 5.7% (*n* = 24), *p* = 0.002) and for shorter duration (42.9% (*n* = 12) vs. 23.6% (*n* = 99), *p* = 0.023 treat iDDVT less than 3 months).

Muscular vein(s) thrombosis are sometimes considered as not ‘true’ DVT [18], although data from the CACTUS RCT, the OPTIMEV cohort and from some meta-analyses suggest that their risk of VTE recurrence is similar to that of the more ‘traditional’ deep calf veins DVT [4, 7, 19, 20]. In our survey, 80% of respondents stated that iDDVT anatomical location does not influence their management and this attitude is consistent across countries.

In terms of knowledge gap, a majority of respondents supported an RCT comparing prophylactic vs. therapeutic anticoagulation, rather than comparing anticoagulation to a placebo or no anticoagulation (Table 1). The absence of a no active treatment arm may be explained by the fact that, in routine clinical practice, respondents generally treat iDDVT with therapeutic anticoagulation. Health care professionals seem more interested in de-escalating iDDVT anticoagulant intensity rather than on holding on any anticoagulant treatment. Two ongoing RCTs using rivaroxaban and apixaban, with and without initial loading dose may provide important information on the optimal anticoagulant dose regimen for iDDVT (ClinicalTrials.gov NCT04967573 and NCT04967573).

Our study has a number of limitations. First, our results are based on self-reporting rather than direct audit of clinical practice and actual practice may, in some cases, differ. Second, our survey population mainly consists of, French, Canadian and Swiss healthcare professionals (90.3%). Our results cannot be extrapolated to other countries. Also, we could not account for multiple respondents from the same hospitals. Our response rate cannot be assessed precisely since many French, Canadian and Swiss healthcare professionals belong to at least 2 participating organizations. It is estimated that 20% of SFMV members, 35% of Thrombosis Canada members and 18% of members of SSA participated to the study. Such participation rates limit generalizability but compares favorably to other similar email international survey studies [21] although it is lower than when healthcare professionals are contacted directly by phone (up to 64%) [22]. Additionally, we lack precise data on the proportion of physicians, nurses, and pharmacists who responded to the survey, as this information was only collected in the Canadian version of the questionnaire. However, where this data was available, the majority of respondents (over 93%) were physicians. Last, to limit the time required to complete the survey, we did not separate questions based on iDDVT etiology (unprovoked vs. provoked vs. cancer-related…). To our knowledge this is the first international survey focusing on the management of iDDVT. It provides complementary results to those of the international RIETE registry, such as the management of muscular DVT as well as healthcare professionals suggestions in terms of research.

In this international survey mostly of French, Canadian and Swiss healthcare professionals, in case of suspected DVT, the vast majority of respondents routinely investigate the calf veins. When IDDVT is detected, most respondents treat their patients with standard therapeutic doses of anticoagulants for 3 months and muscular and deep calf vein DVT are treated the same. Future studies on iDDVT should focus on comparing prophylactic to therapeutic anticoagulation.

## Electronic supplementary material

Below is the link to the electronic supplementary material.

Supplementary Material 1

## Data Availability

No datasets were generated or analysed during the current study.
