# Supplementary material for: Treatment of isolated distal deep vein thrombosis: an international survey of healthcare professionals
Source: J Thromb Thrombolysis. 2025 Apr 11;58(5):657–62. doi: 10.1007/s11239-025-03091-5 (PMC12148997; doi:10.1007/s11239-025-03091-5)
Supplement: Supplementary file 1 — Supplementary Material 1 [file 11239_2025_3091_MOESM1_ESM.pdf]

Additional Table 1: Survey questions

Question number	Question	Answer options
1	At your institution, for patients with clinically suspected lower limb DVT, does the ultrasound protocol include imaging of calf veins (i.e. below/distal to the calf trifurcation)?	- Never<br>- < 50% of the time<br>- > 50% of the time<br>- Always
2	When you see a patient with an acute symptomatic distal DVT and that there is no bleeding concerns, how often do you treat these patients with anticoagulants?	- I am not involved in the treatment of patients with distal DVT [1]<br>- 0-25% of the time<br>- 26% - 50% of the time<br>- 51% - 75% of the time<br>- > 75% of the time
3	How often do you treat patients with an acute distal symptomatic DVT?	- At least once a week<br>- Once a month<br>- Once every 3 months<br>- Less than once every 3 months
4	When you manage an acute symptomatic distal DVT patient, which dose of anticoagulants do you usually use?	- I never treat patients with distal DVT with anticoagulants<br>- Prophylactic doses (i.e. apixaban 2.5mg BID, rivaroxaban 10mg daily, enoxaparin 40mg sc daily…)<br>- Intermediate doses (i.e. higher than prophylactic doses and lower than therapeutic doses, rivaroxaban 15mg daily, enoxaparin 1 mg/Kg sc daily etc…)<br>- Standard therapeutic doses (i.e. same as those used for patients with proximal DVT and /or PE)<br>- Therapeutic doses of DOAC, but without initial higher dose (apixaban 10mg BID for first 7 days or rivaroxaban 15mg BID for first 21 days) if apixaban or rivaroxaban used, or without initial 5-7 days of parenteral anticoagulation if edoxaban or dabigatran used
5	For how long do you usually treat patients with acute distal symptomatic DVT?	- Less than 6 weeks<br>- 6 weeks<br>- 3 months<br>- More than 3 months
6	Do you manage symptomatic muscular DVT (involving soleal or gastrocnemius veins) the same way you manage symptomatic deep calf vein DVT (involving peroneal or posterior tibial veins)?	- Yes<br>- No [2]
7	If you do not manage symptomatic muscular DVT, the same way you manage symptomatic deep calf vein DVT, how management differs?	- I do not treat symptomatic muscular DVT with anticoagulants[3]<br>- I treat symptomatic muscular DVT with lower doses of anticoagulants than symptomatic deep calf DVT?***<br>- I treat symptomatic muscular DVT with shorter durations of anticoagulants than symptomatic deep calf DVT<br>- I treat symptomatic muscular DVT with lower doses of anticoagulants and for shorter duration than symptomatic deep calf DVT
8	If you treat muscular DVT with shorter duration of anticoagulant, how long do you treat them?	- 10 days<br>- 6 weeks<br>- Other, please explain
9	If a new therapeutic trial was planned for the treatment of patients with isolated, acute, distal, symptomatic DVT, what should be the comparator groups?	- Placebo/no anticoagulation vs. prophylactic dosing<br>- Placebo/no anticoagulation vs. full treatment dosing<br>- Prophylactic dosing vs. full treatment dosing anticoagulation<br>- Other

DVT: deep venous thrombosis;

[1] If this answer is selected, participants are not asked to answer questions 4-8; [2] If this answer is selected, participants are not asked to answer questions 7-8; [3] If this answer is selected, participants are not asked to answer question 8
